# Characterization of liquid–liquid phase separation using super-resolution and single-molecule imaging

**DOI:** 10.52601/bpr.2022.210043

**Published:** 2022-02-28

**Authors:** Hongchen Zhang, Shipeng Shao, Yujie Sun

**Affiliations:** 1 State Key Laboratory of Membrane Biology & Biomedical Pioneering Innovation Center (BIOPIC) & School of Future Technology, Peking University, Beijing 100871, China; 2 School of Life Sciences, Peking University, Beijing 100871, China; 3 Beijing Institute of Heart Lung and Blood Vessel Disease, Beijing Anzhen Hospital, Capital Medical University, Beijing 100029, China

**Keywords:** Liquid–liquid phase separation, Super-resolution imaging, Single-molecule imaging

## Abstract

Liquid**–**liquid phase separation (LLPS) is an emerging phenomenon involved in various biological processes. The formation of phase-separated condensates is crucial for many intrinsically disordered proteins to fulfill their biological functions. Using the recombinant protein to reconstitute the formation of condensates *in vitro* has become the standard method to investigate the behavior and function of LLPS. Meanwhile, there is an urgent need to characterize the LLPS in living cells. Importantly, condensates formed through LLPS at physical relevant concentrations are often smaller than the optical diffraction limit, which makes precise characterization and quantification inaccurate due to the scatter of light. The booming development of super-resolution optical microscopy enables the visualization of multiple obscured subcellular components and processes, which is also suitable for the LLPS research. In this protocol, we provide step-by-step instructions to help users take advantage of super-resolution imaging to depict the morphology and quantify the molecule number of endogenous condensates in living cells using RNA Pol II as an example. This streamlined workflow offers exceptional robustness, sensitivity, and precision, which could be easily implemented in any laboratory with an inverted total internal reflection microscope. We expect that super-resolution microscopy will contribute to the investigation of both large and tiny condensates under physiological and pathological conditions and lead our understanding of the mechanism of LLPS to a higher and deeper layer.

## INTRODUCTION

**Figure 1:**
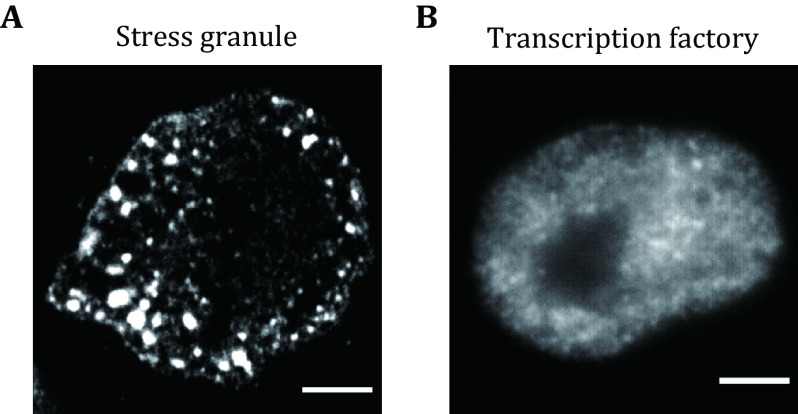
Both giant and tiny condensates co-exist in living cells. **A** Representative fluorescence images of stress granules in HeLa cells under heat shock conditions (42 °C, 0.5 h). Stress granules were labeled using the antibody against G3BP1. Scale bars, 5 μm. **B** Representative fluorescence images of transcription factory in HeLa cells. Transcription factories were labeled using the antibody against RNA Pol II S2 phosphorylation. Scale bars, 2 μm

In addition to canonical membrane-bound organelles, such as endoplasmic reticulum (ER) and mitochondria, cells also take advantage of many membrane-less organelles to compartmentalize and concentrate specific molecules, including nucleolus (Feric* et al.* 2016), Cajal bodies (Razin and Gavrilov 2020), stress granules (Guillen-Boixet* et al.* 2020), and P-bodies (Protter* et al.* 2018). These structures play diverse roles in various biological processes and are also increasingly implicated in protein aggregation diseases (Tsang* et al.* 2020). The underlying mechanism that assembles membrane-less organelles is found to be liquid–liquid phase separation (LLPS). LLPS has been recognized as a novel mechanism in organizing cellular structure and functions. The driving force for LLPS is normally based on weak, transient, and multivalent interactions, including interactions between proteins with multiple repeat domains and a long stretch of intrinsically disordered regions (IDRs) (Shin and Brangwynne 2017). Dependent on the interaction strength, the size of the liquid droplets varies from several hundred nanometers to several micrometers (Zhang *et al*. 2022). For giant droplets, such as stress granules, conventional wide-field fluorescence imaging can easily distinguish and characterize them (Fig. 1A). However, for small droplets that are beyond the diffraction limit, conventional imaging techniques are unable to fulfill the purpose for precise quantification (Fig. 1B). Although multiple techniques such as attaching Cry2 to IDR have been developed to amplify condensate sizes of LLPS (Shin* et al.* 2017), it is more appropriate to study the condensate under physiological conditions. Super-resolution imaging and single-molecule imaging may shed light on the functional interrogations of intracellular physicochemical parameters at the nanoscale.

### Development of LLPS characterization using super-resolution imaging methods

The emergence of super-resolution microscopy has enabled our understanding of cell biology in a more detailed manner. The single-molecule localization-based super-resolution imaging microscopy (SMLM) is especially powerful due to its high resolution and quantitative property. SMLM is fundamentally based on the fact that the spatial coordinates of single fluorophores or emitters can be determined with high precision if nearby fluorophores do not emit in the same frame. Photo-activated localization microscopy (PALM) and stochastic optical reconstruction microscopy (STORM), as two commonly used SMLM techniques, were invented in 2006 (Betzig* et al.* 2006; Hess* et al.* 2006; Rust* et al.* 2006). With a lateral spatial resolution of 10–30 nm and axial resolution of about 50 nm, these techniques have been applied in the analysis of many biological studies, including phase-separated condensates (Azaldegui* et al.* 2021; Fasciani* et al.* 2020). SMLM allows the localization of every single molecule within small condensates that is smaller than the optical diffract limit. Additionally, SMLM also provides the dynamic information of molecules in LLPS condensates, which is extremely useful to characterize the diffusion behaviors of molecules in the condensed phase and dilute phase. For example, recent investigations in bacteria uncovered that membrane-less organelles formed through LLPS might play a crucial role in the organization of bacterial cells. Super-resolution imaging and single-molecule imaging technique have been used to characterize the biochemical functions and assembly mechanisms of these compartments within diffraction-limited foci in bacteria (Azaldegui* et al.* 2021). Moreover, the role of LLPS in transcription regulation has been reported extensively in recent years (Boija* et al.* 2018; Cai* et al.* 2019; Guo* et al.* 2019; Lu* et al.* 2018, 2020). However, it is challenging to directly observe the formation of LLPS condensate during transcription because these condensates are formed through weak and multivalent interaction between the transcription apparatus of low concentration. Using STORM, the detail about how mixed-lineage leukemia 4 (MLL4) contributes to the assembly of transcriptional condensates through LLPS has been reported (Fasciani* et al.* 2020).

Here, we provide a detailed protocol about how to label, image, and analyze LLPS condensates using SMLM. We expect that this protocol will facilitate the investigation of the morphology, material properties, and dynamics of LLPS condensates.

### Applications and advantages of the protocol

This tutorial protocol provides a step-by-step guide of the imaging of LLPS condensates using STORM from sample preparation to images analysis. Using this protocol, we can depict the morphology and quantify the molecule number of endogenous condensates in living cells with high stability, precision, and reproducibility. Although we used RNA Pol II as an example here, this method can be easily adapted to image other protein condensates in cells in the same way.

### Limitations of the protocol

The method for super-resolution imaging offers a high spatial resolution but is limited in the throughput. It only permits the imaging of one type of protein condensate at the acquiring time. The imaging process also takes a long time to capture enough single molecules, which also limits the throughput. Owing to the principle of STORM, it can not be used to address the molecular dynamics within the condensates in fixed cells. But single molecules tracking in living cells can solve this problem.

## MATERIALS AND EQUIPMENT

### Biological materials

• Adherent Cell. We have used human HeLa S6 and other mammalian cell lines, for super-resolution and single-molecule imaging. The cell lines used should be regularly checked to ensure they are authentic and are not infected with mycoplasma.

### Reagents

• Distilled, deionized H_2_O (ddH_2_O, Promega, cat. no. P1197)

• 10% (*v*/*v*) Triton-X100 (Sigma-Aldrich, cat. no. X100)

• Primary antibody to an epitope of interest, *e*.*g*., recombinant Anti-RNA polymerase II CTD repeat YSPTSPS (phospho S2) antibody [EPR18855-87]-ChIP Grade (abcam, cat. no. ab238146, dilution 1:100), anti-G3BP1 (CST, E9G1M, Rabbit mAb, cat. no. #61559, 1:100 dilution)

• Secondary antibodies, Rabbit IgG (H+L) Cross-Adsorbed Secondary Antibody-Alexa Fluor 647 (ThermoFisher, cat. no. A-21244, dilution 1:100)

• Growth medium DMEM (GIBCO, cat. no. 11995065) supplemented with 10% (*v*/*v*) FBS, 1% penicillin and streptomycin (ThermoFisher, cat. no. 10378016).

• Bovine serum albumin (Sigma-Aldrich, cat. no. A1933)

• PBS (ThermoFisher, cat. no. 10010002)

• Lipofectamine 3000 (ThermoFisher, cat. no. L3000015)

• Trypsin-EDTA (ThermoFisher, cat. no. 25300120)

• Glucose oxidase (Sigma-Aldrich, cat. no. 345386)

• Catalase (Sigma-Aldrich, cat. no. C3515)

• Glucose (Sigma-Aldrich, cat. no. D9434)

• Dulbecco’s PBS (DPBS, Sigma-Aldrich, cat. no. D8537-1l)

• Oxygen scavenger system：consists of 0.5 mg/mL glucose oxidase, 40 μg/mL catalase, 10 % (*w*/*v*) glucose in phosphate buffer (pH 7.4)

• Paraformaldehyde (PFA, TAAB, cat. no. P001)

**[CAUTION!]** PFA is toxic, irritant, flammable, and corrosive. It is hazardous on skin or eye contact or inhalation. Wear gloves and use it in a fume hood.

• Glutaraldehyde (GA, Sigma-Aldrich, cat. no. G5882)

**[CAUTION!]** GA is toxic, irritant, flammable, and corrosive. It is hazardous on skin or eye contact or inhalation. Wear gloves and use it in a fume hood.

• Poly-L-lysine, succinylated (Sigma-Aldrich, cat. no. P3513)

• Qdot 655 ITK Amino (PEG) Quantum Dots (ThermoFisher, cat. no. Q21521MP)

### Equipment

• Standard UV-visible spectrophotometer (Thermo Scientific, NanoDrop2000,)

• Inverted fluorescence microscope (Olympus IX-71)

• Standard equipment for mammalian cell culture with CO_2_ incubator (ThermoFisher)

• Horizontal rotator (ThermoFisher)

• Aspirator

• Coverslips (ThermoFisher, cat. no. S17525B)

• Six-well plate

• Microcentrifuge

• Microwave oven

• 35 mm glass-bottom Petri-dishes with 10 mm Bottom Well (CellVis, cat. no. D35-10-1.5-N)

• PES Syringe Filter, 0.22 um, 13 mm (BioVision, cat. no. M4329)

• Orbital shaker (Biosan OS-10)

• Pipette Pasteur

• Oil-immersion objective (100×, PlanApo 1.45 NA, Olympus)

• Irradiation lasers: 647 nm (Cube 640-100C, Coherent), 561 nm (Sapphire 568 LP, Coherent), 488 nm (Sapphire 488 LP, Coherent), 405 nm (Cube 405-100C, Coherent)

• Acousto-optical tunable filter (AOTF, AAOptics) for laser line selection

• Delay and pulse generator (Stanford Research Systems)

• Neutral density filter to adjust laser intensity (Edmund Optics)

• Single-mode fiber (Linos)

• Fiber coupler (Linos)

• Band-pass / long-pass filters to spectrally separate fluorescence signal (Semrock, Chroma, AHF Analysentechnik)

• RazorEdge 647 Long-pass filter

• Laser clean-up filter (Semrock, Chroma, AHF Analysentechnik)

• Electron multiplying charged-coupled device camera (Ixon; Andor)

• Large Chip (Ixon DU897, 512 × 512 pixels with 16 μm pixel size, Andor)

• Software to control camera (Andor Solis, Andor)

### Buffer

• Fixation buffer (make freshly)

PFA: [Stock] = 8%, [Final] = 2.8%,* V* = 1.75 mL

GA: [Stock] = 8%, [Final] = 0.04%,* V* = 0.0025 mL

1× PBS: *V* = 3.25 mL

• PBST buffer (make freshly)

Triton X-100: [Stock] = 10%, [Final] = 0.5%,* V* = 0.5 mL

• Blocking buffer (make freshly)

BSA: [Stock] = 10%, [Final] = 5%,* V* = 1 mL

Triton X-100: [Stock] = 10%, [Final] = 0.5%,* V* = 0.1 mL

1× PBS: *V* = 0.9 mL

• Standard imaging buffer (STIB) for STORM imaging

Tris/HCl (pH 8.0): [Stock] = 1 mol/L, [Final] = 100 mmol/L,* V* = 3 mL

NaCl: [Stock] = 5 mol/L, [Final] = 20 mmol/L,* V* = 0.12 mL

Glucose: [Stock] = 30%, [Final] = 10%,* V* = 10 mL

ddH_2_O: *V* = 16.88 mL

**[CRITICAL STEP]** Filter the buffer using a 0.22 μm filter to remove bacterial and dust. Vacuum to remove the O_2_ in the buffer.

Glucose oxidase: [Final] = 60 mg/mL,* W* = 120 mg

• GLOX (glucose oxidase and catalase) (4 °C, 1−2 weeks)

Catalase: [Final] = 6 mg/mL,* W* = 12 mg

Glycerol: [Stock] = 80%, [Final] = 40%,* V* = 1 mL

1× PBS: *V* = 1.5 mL

**[CRITICAL STEP]** Centrifuge at maximum speed for 1 min. Use the yellow supernatant for imaging buffer. Aliquot 200 μL. The color of the mixture is determined by different Lot. No. of the catalase.

• Final STORM imaging buffer (make freshly)

STIB: *V* = 1.96 mL

β-ME: [Stock] = 14.4 mol/L, [Final] = 14.4 mmol/L,* V* = 0.02 mL

GLOX: *V* = 0.02 mL

**[CRITICAL STEP]** Make fresh and use within 30–60 min.

### Software

• Image J, free download at https://imagej.net/software/fiji/

• ThunderSTORM (Ovesny* et al.* 2014), free download at https://zitmen.github.io/thunderstorm/

• MATLAB, available at https://ww2.mathworks.cn/products/matlab.html

• SR-Tesseler (Levet* et al.* 2015), free download at https://github.com/flevet/SR-Tesseler/releases/

• Rstudio, free download at https://www.rstudio.com/

• The customed MATLAB scripts used in this protocol can be downloaded from https://zenodo.org/record/5906981#.YfFmov77QuU

## PROCEDURE

### Step 1: Sample preparation

For the labeling of droplets in fixed cells with antibodies (Alexa 647), follow the steps in Step 1.1 (RPB1 as an example). For labeling of droplets in living cells with a fluorescent protein (mMaple3 as an example), see Step 1.2.

#### Step 1.1 Fixed cells with antibodies

Step 1.1.1: One day before experiments, passage cells into 35 mm glass-bottom Petri-dishes or chambered cover glass.

Step 1.1.2: Wash cells twice with 1× PBS, add PFA to a final concentration of 4% (*v*/*v*) and incubate for 10 min at room temperature (RT, 20 °C) to fix the cells.

**[CAUTION!]** Wear appropriate protective equipment and avoid contact with skin or eyes.

Step 1.1.3: Wash the cells twice with 1× PBS to remove residual PFA. Aspirate PBS and add 200 μL Triton X-100 at a concentration of 0.5% (*v*/*v*) in 1× PBS to each dish, then incubate again at RT for 10 min.

Step 1.1.4: Rinse twice with 1× PBS.

Step 1.1.5: Prepare the blocking buffer containing 5% (*v*/*v*) BSA and 0.5% (*v*/*v*) Triton X-100 in 1× PBS. Add 200 μL blocking buffer to each chamber and incubate for 30 min at room temperature (20 °C).

**[? TROUBLESHOOTING]**

Step 1.1.6: Remove the blocking solution and dissolve antibody in blocking buffer containing 5% (*v*/*v*) BSA and 0.5% (*v*/*v*) Triton X-100 in 1× PBS at different concentrations, (1:100, 1:200, 1:500 and 1:1000). Incubate for 1–2 h at room temperature (20 °C) or 4 °C overnight.

**[? TROUBLESHOOTING]**

Step 1.1.7: Exchange the antibody solution in each chamber with PBST and incubate for 5 min at room temperature (20 °C). Repeat this step twice.

Step 1.1.8: Add secondary fluorescently labeled antibody dissolved in blocking buffer (200 μL) to each sample at different dilutions and incubate for 30–60 min in the dark at room temperature (20 °C).

**[? TROUBLESHOOTING]**

Step 1.1.9: Rinse twice with PBS.

Step 1.1.10: Post-fix the cells with fixation buffer.

**[CAUTION!]** Wear appropriate protective equipment and avoid contact with skin or eyes.

Step 1.1.11: Rinse twice with 1× PBS.

#### Step 1.2 Live cells with fluorescent proteins

Step 1.2.1: Block the surface of the dish with the poly-L-lysine solution for 30–60 min.

Step 1.2.2: Wash the dishes with 1× PBS three times.

Step 1.2.3: Transfer cells into a poly-L-lysine treated dish one day before transfection.

Step 1.2.4: Transfect cells with a vector encoding the protein of interest fusion with a fluorescent protein (mMaple3) using Lipofectamine 3000 according to the protocol given by the supplier and incubate cells overnight in a growth medium.

**[? TROUBLESHOOTING]**

Step 1.2.5: Wash cells twice with 1× PBS, add PFA to a final concentration of 4% (*v*/*v*) and incubate for 10 min at room temperature (RT, 20 °C) to fix the cells.

Step 1.2.6: Wash the cells twice with 1× PBS to remove residual PFA.

### Step 2: Microscope setup

Step 2.1: Turn on all the lasers, adjust to a low laser power (1–2 mW), and block beams using appropriate shutters in the excitation path. Allow gas lasers to warm up for 20 min.

**[CAUTION!]** Avoid direct laser eyes and wear goggles.

Step 2.2: Measure laser power in focus after the polychromic mirror before entering the objective. Determine homogeneously irradiated area by imaging the fluorescence signal of a fluorophore or QD solution (~10^−8^ mol/L) on the EMCCD camera to calculate the irradiation intensity in kW/cm^2^.

Step 2.3: Set the experimental procedure. Adjusting the storage path, naming the file, adjusting the EMCCD procedure, and setting the control program of the shutter.

Step 2.4: Remove the PBS, and 10 µL of silica beads (resuspend in PBS 1:200 0.5% (*v*/*v*)) were added per sample and incubated at RT for 0.5–2 h.

Step 2.5: Violent shock for 5 min and remove the superfluous silica beads. Ideally, there are 3–10 silica beads in a 256 × 256 pixel view.

Step 2.6: Use 2 mL final STORM imaging buffer to replace the PBS. For PALM imaging with a fluorescent protein, use cold PBS instead of STIB.

**[CRITICAL STEP]** STIB should be made fresh and used within 60 min. If the image acquisition time is longer than 60 min, re-prepare the fresh STIB.

**[? TROUBLESHOOTING]**

Step 2.7: Place experimental samples, use the oil-immersion objective and adjust the laser according to Table 1.

**Table 1:** Ideal working and output laser power

Laser	Laser working power	Measure laser power
647	1000 mW	300 mW
561	1000 mW	230 mW
405	MAX	10 µW
488	30 mW–Low	130 µW

### Step 3: Image acquisition

#### Step 3.1: Cell selection

Adjust the focus so that the sample is in the focal plane, move the mirror toward TIRF geometry at a certain point, and turn on the infrared focus lock. To find the optimal conditions for imaging, the selected cells should have ideal structures and at least three silica beads around.

**[? TROUBLESHOOTING]**

#### Step 3.2: Data acquisition

For the labeling of droplets in fixed cells with antibodies, follow the steps in Step 3.2.1 (with RPB1 as an example). For labeling of droplets in living cells with a fluorescent protein (mMaple3), see Step 3.2.2.

Step 3.2.1 Antibody-labeled sample

Step 3.2.1.1: Capture the conventional image in the 647 nm channel. It is ideal to capture 100–200 frames of conventional images to get the average image.

Step 3.2.1.2: Before performing 647–brightfield experiments, increase irradiation intensity to the max (1000 mW) for a few minutes to transfer the majority of fluorophores to the OFF state. Ideally, only a sparse subset of fluorophores resides in the ON state.

**[? TROUBLESHOOTING]**

Step 3.2.1.3: Capture the 647 STORM image without the 405 nm laser. Adjust the Laser power so that the single-molecule signal can be resolved and the photo-bleach effect is minimized.

Step 3.2.1.4: Remove the sample, and wash with 1× PBS three times. For long-term storage, wash with ddH_2_O three times. Remove the liquid and store the dish at 4 °C.

The sequence and exposure conditions for antibody-labeled proteins are listed in Table 2.

**Table 2:** The sequence and exposure conditions for antibody-labeled proteins

Laser	Imaging mode	EMCCD exposure	EMCCD exposure compensation	Frames
647 nm	Conventional	30 ms	30	200
647 nm–brightfield	Conventional	10 ms	30	200
647 nm	STORM	10 ms	30	50,000

Step 3.2.2 Fluorescent protein-labeled sample

Step 3.2.2.1: Capture the conventional image in the 488 nm channel. It is ideal to capture 100–200 frames of conventional images to get the average image.

Step 3.2.2.2: Before performing 561 nm brightfield experiments, increase irradiation intensity to the max (1000 mW) for a few minutes to bleach the majority of activated fluorescent proteins.

**[? TROUBLESHOOTING]**

Step 3.2.2.3: Capture the 561 nm STORM image with the 405 nm laser simultaneously. Adjust the 561 nm laser power so that the single-molecule signal can be resolved and the photo-bleach effect is minimized.

**[CRITICAL STEP]** For the activation of fluorescent protein using the 405 nm laser, increase the laser power gradually to ensure the approximate number of molecules was activated during image acquisition.

Step 3.2.2.4: Remove the sample, and wash with 1× PBS three times. For long-term storage, wash with ddH_2_O three times. Remove the liquid and store the dish at 4 °C.

The sequence and exposure conditions for fluorescent protein-labeled proteins are listed in Table 3.

**Table 3:** The sequence and exposure conditions for fluorescent protein-labeled proteins

Laser	Imaging mode	EMCCD exposure	EMCCD exposure compensation	Frames
488 nm	Conventional	30 ms	90	200
561 nm–brightfield	Conventional	30 ms	90	200
561 and 405 nm	STORM	30 ms	90	50,000

### Step 4: Image analysis and quantification

**Figure 2:**
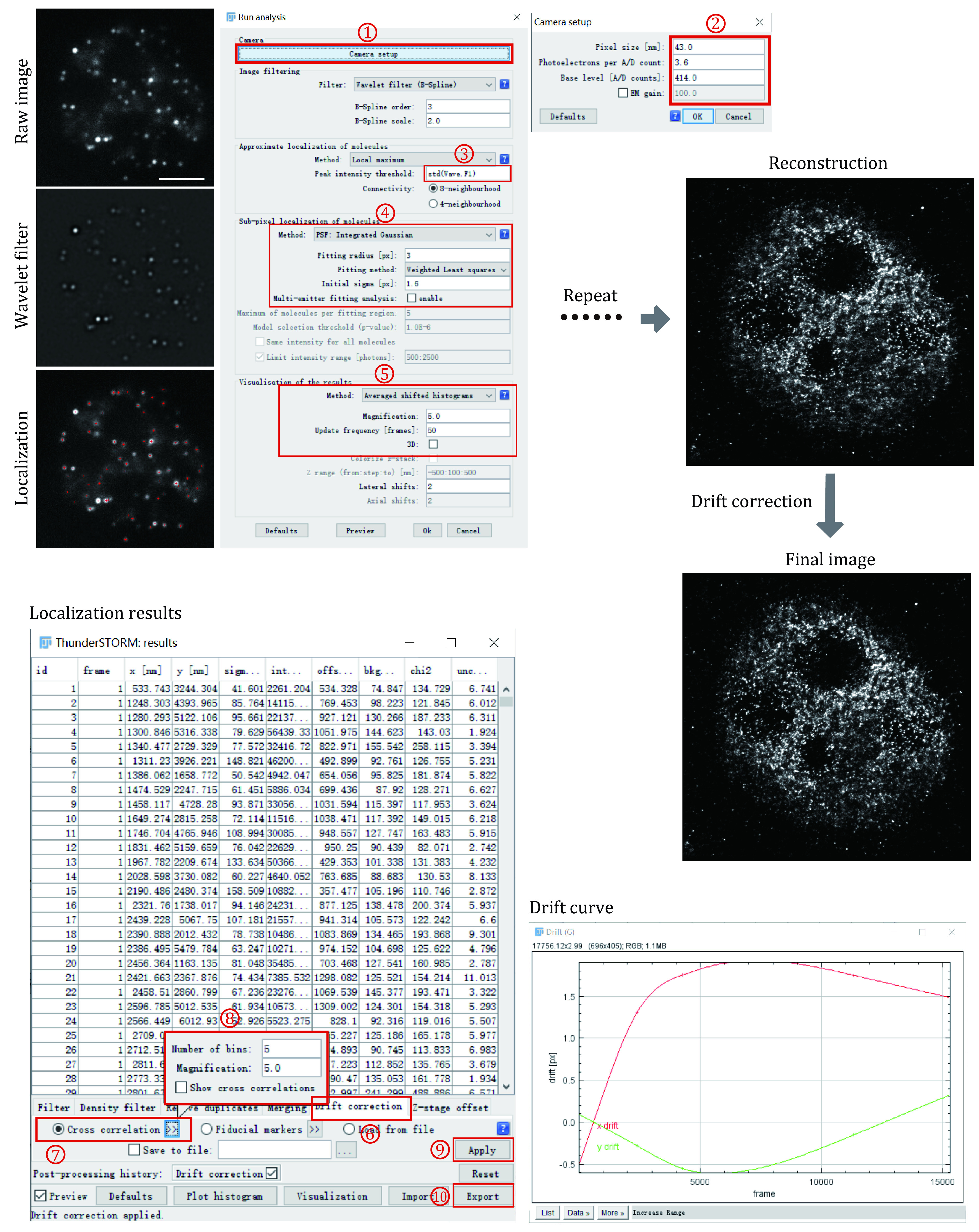
Data processing for super-resolution images of small condensates. Each frame of the single-molecule raw image was first filtered using a wavelet filter and then localized using Gaussian fitting. This process was repeated for each frame until all the frames have been processed. After single-molecule localization and image reconstruction, drift correction using cross-correlation was applied to correct the drift during image acquiring

Use Image J Plugin ThunderSTORM to automatedly process, analyze, and visualize data acquired by single-molecule localization microscopy methods such as PALM and STORM (Fig. 2).

Step 4.1: Install ImageJ and download the latest version of ThunderSTORM (https://zitmen.github.io/thunderstorm/). For installation, copy the downloaded file into ImageJ's plugin subdirectory and run ImageJ.

Step 4.2: Load the single-molecule localization dataset through ImageJ File→Open or direct drag and drop the data file from the file explorer to ImageJ.

Step 4.3: Set the camera pixel size, the conversion factor between photons and digital units, the base-level offset of the camera digitizer, and EM gain of the camera through Plugins→ThunderSTORM→Camera setup. These parameters are important for fitting and estimation of localization accuracy.

Step 4.4: With the data opened in ImageJ windows, select Plugins→ThunderSTORM→Run analysis to start the analysis process. The default options provided by this plugin can achieve very satisfactory results on various samples and datasets. If we want to get a better result or we have a special dataset that is different from the default parameter, there is a lot of freedom to set the processing parameters. Then press OK to start the image localization process. During the data analysis, an instant preview of the rendering image result is shown, which keeps updating during the whole analysis process.

Step 4.5: The visualized super-resolution image and the table of localized molecules will appear after the analysis process. We then correct the drift using cross-correlation. A drift curve will appear when the correction is finished. Importantly, it is always suggested to perform the drift correction for STORM images as the image acquisition takes tens of minutes to hours, which will cause motion blur if keep uncorrected.

**[? TROUBLESHOOTING]**

Step 4.6: Visualize the super-resolution image through Plugins→ThunderSTORM→Visualization. This will create a new, high-resolution image based on the previously obtained sub-diffraction molecular coordinates. Several visualization methods have been implemented in this plugin and choose one suitable for our datasets. Save the super-resolution images in any image format (TIF, JPEG, PNG) provided by ImageJ. If we close the data table during the image process accidentally, we can reopen it through Plugins→ThunderSTORM→ Import/Export→Show results table.

Step 4.7: Exporting the table of results in various file formats for further analysis, such as CSV, XSL, XML, YAML, JSON, Google protocol buffer, and Tagged Spot File (TSF) format. The exported results can be post-processed and visualized by other localization software. Plugins→ThunderSTORM→Import/Export→Export results.

### Step 5: Condensate identification and segmentation

**Figure 3:**
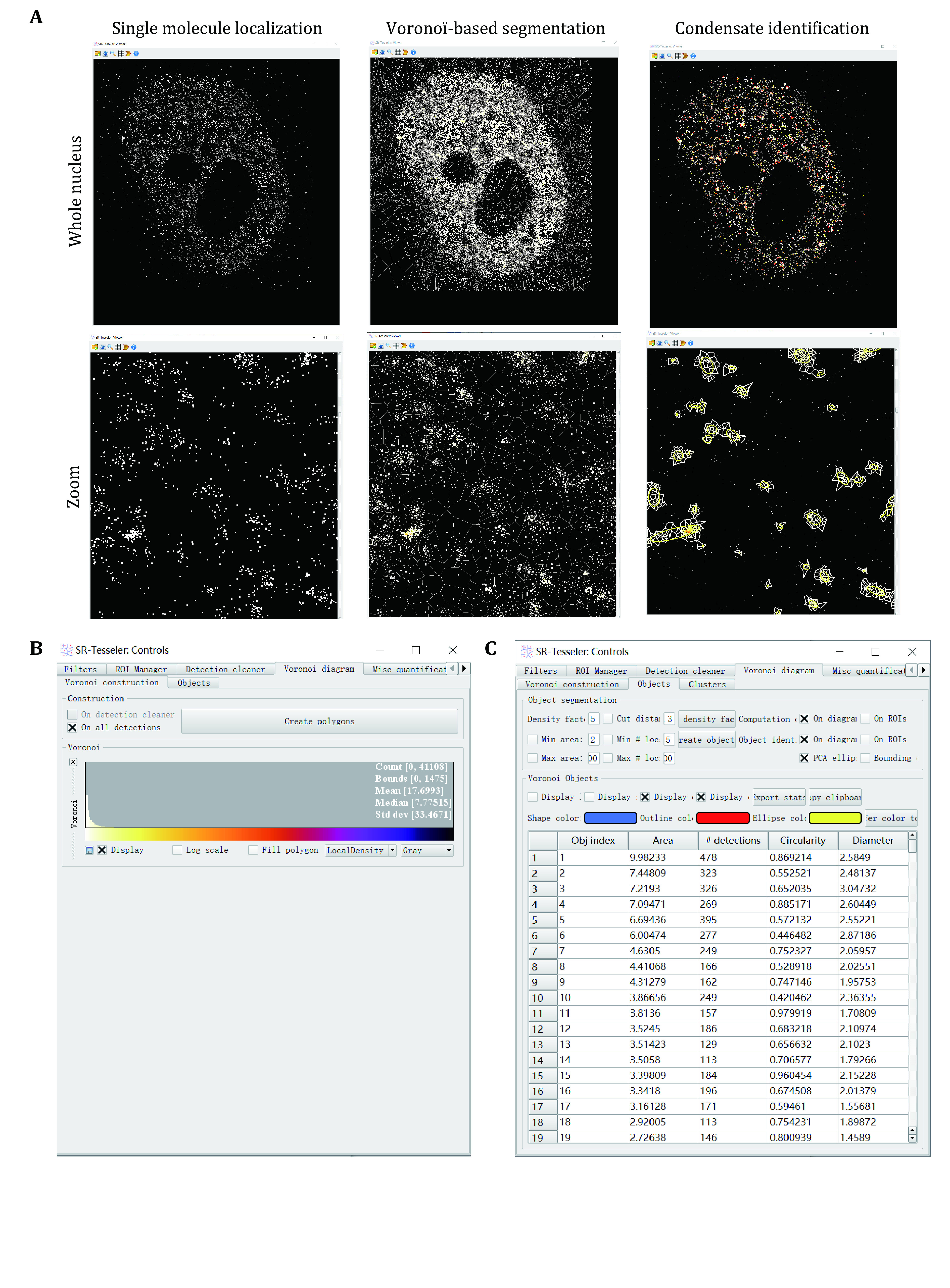
Condensate identification and quantification using super-resolution imaging. **A** Voronoï-based segmentation of condensates using SR-Tesseler. Zoomed images were displayed at the bottom for more detailed information. Every single molecule has a polygon defined by its neighboring molecules. Edges of Voronoï polygons are located equidistant from the nearest two molecules. When a new molecule is added, this bisector is cut by the bisectors computed between the old molecules and the new ones. Each new molecule was computed to plot the Voronoï diagram repeatedly until all the molecules were counted. Then a threshold relative to the average localization density was set to segment the clusters of molecules within one condensate. **B** Create polygons in SR-Tesseler. **C** Parameters used to define condensates in SR-Tesseler

We use SR-Tesseler to identify and segment LLPS condensates from PALM and STORM images (Fig. 3).

Step 5.1: Download the latest version of SR-Tesseler and install it following the instruction.

Step 5.2: Transfer the data exported from ThunderSTORM (csv format) to ASCII file in the following format.Header: [Nbplanes, Nbdetections]; List of detections; [x y intensity frame sigma] (sigma is optional).

**[CRITICAL STEP]** For the comparative of condensates between the different groups, such as localization per condensate, condensate number per cell, it is critical to resample the single-molecule dataset to ensure each group has the same localization number. In our RNA Pol II example, we randomly choose 100,000 molecules for each group.

Step 5.3: Launch SR-Tesseler.exe and then two windows will pop up: a Console (for application messages) and a Viewer.

Step 5.4: Click on the open icon to open single-molecule data and select the localization file generated from the previous step.

Step 5.5: When the loading process is finished, the super-resolution image is displayed in the SR-Tesseler Viewer window and a control window with many display options is shown.

Step 5.6: Then click the Create polygon button, the Voronoï diagram can be computed on the initial detection dataset.

Step 5.7: Create the object by adjusting the density factor (of δ), selecting and adjusting the minimum area (in pixel²) and the number of localizations of the objects, and a cut distance (in pixel) if needed.

Step 5.8: Export the object information as an Excel file in the data directory clicking the Export stats button for further analysis.

### Anticipated results

**Figure 4:**
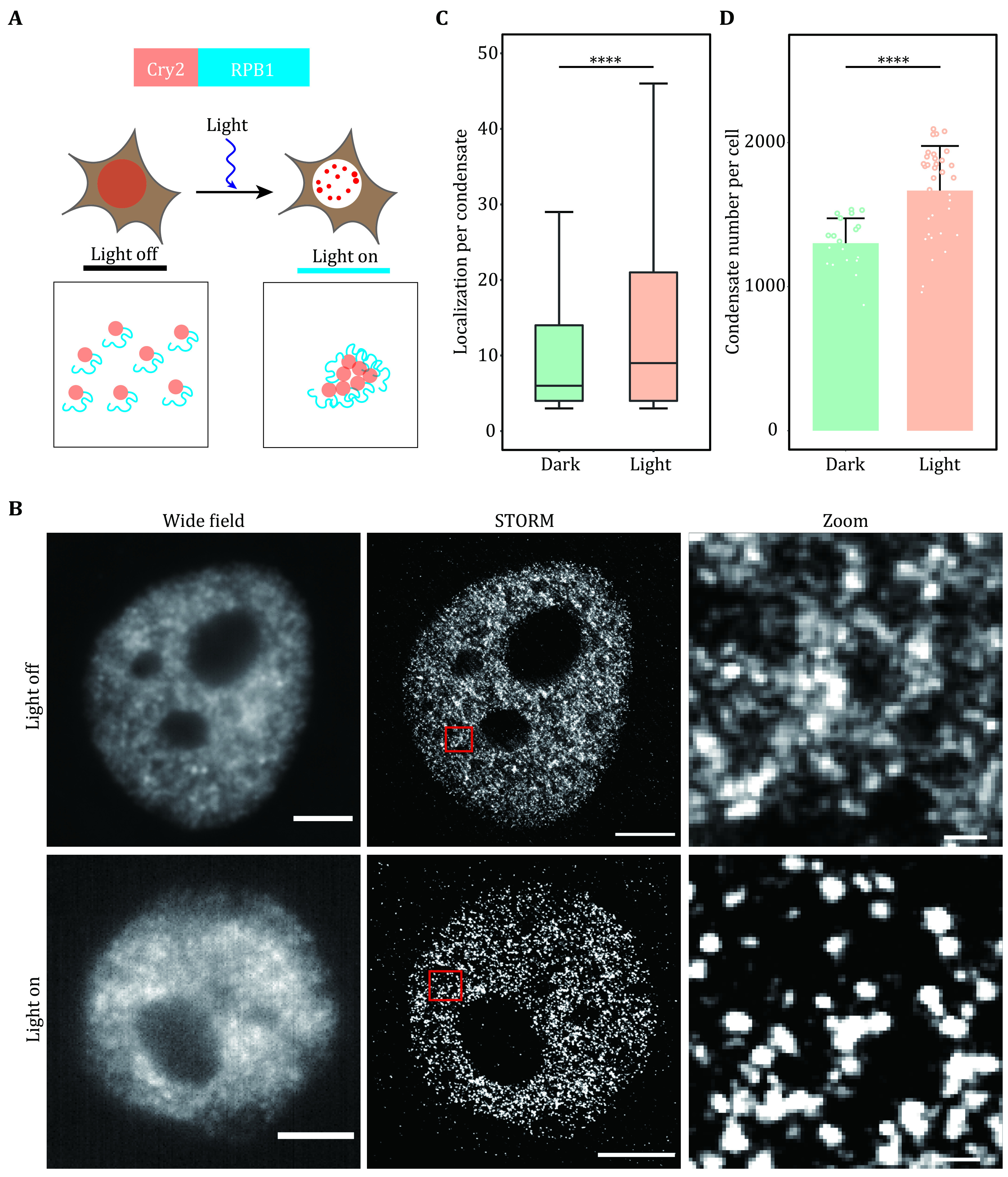
Super-resolution imaging of transcription factory induced by optogenetics. **A** Schematic diagram of light-induced condensation enhancement. The largest subunit of RNA Pol II (RPB1) was fused with Cry2 to induce cluster formation. Blue light activation of Opto-RPB1 leads to rapid phase separation in living cells. **B** Representative wide-field and STORM images of Cry2-RPB1 before and after blue light illumination. The transcription factory was labeled using the antibody against RPB1. Scale bars, 2 μm. Zoomed scale bars, 100 nm. **C**, **D** Cluster analysis of RPB1 under the light on and light off conditions. Localization per condensate (C) and condensate number per cell (D) were shown. Data are presented as mean ± SD. Individual data points correspond to the average value for one cell. *n* = 20 cells for dark condition, 34 cells for light condition pooled from three independent experiments. The paired two-tailed Student’s *t*-test was used to compare the data (C and D). Significant differences are labeled as ****p < 0.0001. Boxplots: 25^th^ to 75^th^ percentiles, median, 1.5× interquartile as whiskers (C)

Generally, after following this protocol, we could get the super-resolution images of LLPS condensates under different conditions with high resolution (Fig. 4A and 4B). Additionally, we can further analyze the statistical information of molecules within condensated from different groups. This information included localization per condensate, condensate number per cell, condensated area (Fig. 4C and 4D).

In the example of light-induced transcription factory formation, we have proved that blue light illumination can enhance RNA Pol II LLPS when RPB1 was fused with light response module Cry2. In the dark state, although we could observe some RPB1 clusters, most of the molecules were dispersed. After light induction, nearly all RPB1 molecules enter the condensate, indicating the power of the super-resolution imaging technique in quantifying the subtle change of tiny condensate in cells.

## TIMING

Step 1: Sample preparation (~6 h)

Step 2: Microscope setup (1–2 h)

Step3: Image acquisition (0.5–2 h for one image)

Step 4: Image analysis and quantification (0.2–0.5 h for one image)

Step 5: Condensate identification and segmentation (~0.5 h for one image)

**[?TROUBLESHOOTING]**

Troubleshooting advice can be found in Table 4.

**Table 4:** Troubleshooting table

Step	Problem	Problem reason	Solution
Step 1.1.5	High background	Blocking missed/failed	Increase the concentration of BSA; Use fresh BSA solution
Step 1.1.6	High background	Artifacts	Modify the blocking protocol and use different fluorescence excitation to check if it is stained non-specifically.
Step 1.1.6	Structure disruption	Over permeabilization	Modify permeabilization protocol
Step 1.1.6	No fluorescence	Fail in permeabilization	Modify permeabilization protocol; Use fresh permeabilization solution
Step 1.1.8	Low intensity	Low antibody titer	Change the secondary antibody; Modify the incubation time
Step 1.1.8	Low intensity	Unsuitable antibody solution conditions	Check pH of antibody solution, and make fresh
Step 1.1.8	High intensity	Non-specifically stained	Add the control experiments ( do not add the primary antibody)
Step 1.2.4	No fluorescence	Fail in transfection	Use cells in a healthy state; Use the efficient transfection reagent
Step 2.6	Dye not blinking	STIB spoil	Use fresh STIB
Step 3.1	Hard to find ideal cells	Sample drift	Verify sample drift using the calibration sample and realign the focal plane
Step 3.1	Hard to find ideal cells	Mismatched immersion oil	Change the immersion oil
Step 3.1	Hard to find ideal cells	The cells are unhealthy	Optimize the cell culture
Step 3.2.1.2	Surplus resides in the ON state	Insufficient irradiation intensity	Increase irradiation intensity
Step 3.2.1.2	Fluorophores do not recover	Only a sparse subset of fluorophores is in the ON state	Reduce the intensity of the readout laser
Step 3.2.1.2	Fluorophores do not recover	Fluorophores are photobleached	Optimize the activation and readout laser
Step 3.2.1.2	Fluorophores do not recover	STIB is unsuitable	Make fresh and use within 60 min Check pH; pH should be in the range of 6–9
Step 4.5	Low resolution	Drift correction missing	Perform drift correction

## Conflict of interest

Hongchen Zhang, Shipeng Shao and Yujie Sun declare that they have no conflict of interest.
